# Current Status and Prospects of Genetic Resources of Native Chickens of Japan

**DOI:** 10.3390/ani15121703

**Published:** 2025-06-09

**Authors:** Hideaki Takahashi

**Affiliations:** National Agriculture and Food Research Organization (NARO), Tsukuba 305-8517, Ibaraki, Japan; takahashi8675@gmail.com; Tel.: +81-80-5497-3127

**Keywords:** native chicken breed, Japanese, efficient conservation, effective use, governance

## Abstract

The Japanese government established Japan Agriculture Standards (JAS) in 1999 for the production of chicken meat using native Japanese chicken breeds, abbreviated as ‘Jidori JAS’, for the effective use of native chickens. “Jidori” means “native chickens” in Japanese. The Jidori JAS has designated the Japanese chicken breeds that were established in Japan and/or imported before the end of the Meiji period (until 1912). According to the Jidori JAS, the percentage of native blood in chickens to be marketed as certified Jidori JAS must be 50% or more. This indicates that the Japanese government has permitted the commercial production of hybrid chickens under the Jidori JAS certification. Jidori JAS was introduced to increase the number of chicks available for fattening and improve the growth performance of Jidori JAS certified production. While farmers have to buy chicks from hatcheries each time they produce poultry, this ensures stable production, and the meat quality of the chicken remains consistent. It should be noted that Jidori JAS certification does not guarantee a specific flavor for Jidori meat products. Currently, marker-assisted selection for growth improvement has been successfully implemented in Japan for several Jidori JAS-certified chickens, enhancing their growth performance.

## Simple Summary

Poultry farming using native breeds has been practiced in many countries, especially in developing and underdeveloped countries. Japan is a highly developed country, yet it retains a number of native chicken breeds, some of which are used commercially under strict guidelines set by the Japanese government. International poultry experts understand, through practical cases within the Japanese practices, how to effectively use native chicken breeds for efficient conservation without directly improving their production traits. However, Japanese practices may not be suitable for rural poultry farming in developing countries that directly use native breeds.

## 1. Introduction

The majority of chicken meat in Japan is produced from broiler chicks supplied by a few international breeding companies [1]. Concurrently, Japan has also sustained a number of native chicken breeds [2,3,4]. Several of these native breeds have been utilized commercially under the strict governance of the Japanese government [5]. The objective of this review is to provide information about the current status and future prospects of the genetic resources of native Japanese chickens, with a focus on their commercial utilization, and to provide information about the successful application of marker-assisted selection for growth improvement in branded chickens using breeds native to Japan.

## 2. History of Japanese Native Breeds of Chicken

Given its status as an island country, Japan is geographically isolated from both the Eurasian continent and other island countries. Therefore, it can be said that there are no indigenous chickens in Japan in the true sense of the term. Consequently, the introduction of different chicken breeds to Japan occurred at various points in time. According to popular belief of Japanese chickens [2,3,4], most of today’s Japanese chicken breeds were established from three original breeds, Jidori, Shokoku, and Shamo. Jidori means native chicken and retains primitive chicken characteristics. Jidori is thought to have been introduced into Japan from China about 2000 years ago. Shokoku, which has long hackle and saddle feathers, is thought to have been introduced into Japan from China between the 8th and 12th centuries. Some varieties of Shokoku were exported from Japan to other countries in the 19th century, and their offspring are known as Phoenix and Yokohama [6,7]. Shamo is thought to be derived from a Malay-type chicken introduced into Japan from Thailand in the 16th or 17th century for cockfighting [2,3,4]. In addition, other types of breeds, such as Tomaru and Ukokkei, were introduced into Japan from China [2,3,4]. During the Edo period (1603–1868), the Tokugawa shogunate implemented a policy of strict isolation, prohibiting foreign trade with the exception of China and the Netherlands from 1635 to 1854. Concurrently, Japan maintained the exchange of correspondence with Korea and the Ryukyu Kingdom (present-day Okinawa Prefecture, Japan). During the period of national trade restrictions, various breeds were bred for the purpose of enhancing specific characteristics. These included an increased ability to crow, enhanced ability for cockfighting, and enhanced esthetic qualities, such as miniature body size, beautiful and distinctive plumage, short legs (Creeper-trait), and long tail feathers. Those with expertise in Japanese chickens define “Japanese native breeds” as chickens that were established prior to the close of the Edo period. Prior to the conclusion of World War I, maritime travel represented the sole means of international travel, and the number of domestic animals in Japan was much smaller than it is today. So, the risk of animals catching infectious diseases was much lower than it is today. In addition, the Edo period is characterized by a period of peace, known as the *Pax Tokugawana*. People could enjoy fancy chickens. After the Meiji period (1868–1912), the number of native Japanese chickens, especially the fancy breeds, declined considerably because people were less interested in owning Japanese chickens and there was a greater demand for chickens for meat and eggs. According to Tsudzuki (2003) [3], based on the narrow definition, there are 40 to 50 breeds known to be native to Japan. Of these, 17 have been designated as national treasures of Japan (Table 1).

After the Meiji period, the number of native Japanese chickens, especially the fancy breeds, declined considerably because people were less interested in owning Japanese chickens, and there was a greater demand for chickens for meat and eggs. With regard to the commercial utilization of native chicken breeds, the key constraints are low meat and egg productivity. This problem has been identified not only in Japan but also on a global scale. In order to address the increase in demand for meat and eggs, breeds developed in Western countries that are characterized by their efficiency of production, such as the Rhode Island Red and the Barred Plymouth Rock, were introduced to Japan. Furthermore, certain breeds were developed during the Meiji period, including Nagoya (a synthetic breed crossed between local chickens from Nagoya City and Chinese Buff Cochin), Mikawa (a synthetic breed crossed between Buff Leghorn, Chinese Buff Cochin, and Nagoya), and Eikoku. The name ‘Eikoku’ is a reference to Great Britain and Ireland. This breed is thought to be a synthetic cross between local chickens and those brought in by ship under the Union Jack. Kumamoto is a synthetic breed developed in Kumamoto Prefecture by crossing local chickens, Eikoku, Buff Leghorn, Chinese Buff Cochin, and Buff Plymouth Rock. Miyaji-dori and Tosa-kukin are thought to be a synthetic cross between local chickens and Black Minorca, and a synthetic cross between local chickens and Chinese Buff Cochin, respectively, developed in Kochi Prefecture.

## 3. Japanese Agricultural Standards for the Production of Chicken Meat, Requiring the Use of Native Japanese Chicken Breeds

### 3.1. Promotion Policy for Native Chicken Breeds by the Japanese Government

In the 1980s, when Japan’s economic development was at its peak, a gourmet boom occurred in Japan, and the deliciousness of native chicken meat was re-evaluated. In order to promote the effective use of native chicken breeds, the Japanese Ministry of Agriculture, Forestry and Fisheries (MAFF) of Japan has established Japan Agriculture Standards (JAS) for the production of chicken meat using native Japanese chicken breeds, abbreviated as Jidori JAS [5].

Jidori JAS [5] has designated the name ‘Zairaisyu’ for Japanese chicken breeds that were established in Japan and/or imported prior to the close of the Meiji period. Jidori JAS designated thirty-eight breeds as Zairaisyu, including Nagoya, Mikawa, Eikoku, Kumamoto, and Miyaji-dori, Barred Plymouth Rock, Rhode Island Red, and Chinese Buff Cochin (Table 2).

Confusingly, the Japanese word ‘Zairaisyu’ means (a) native chicken/chickens. Those familiar with Japanese chickens define “Japanese native breeds” as chickens that were established before the Edo period. It is important to make a clear distinction between breeds established in Japan before the end of the Edo period, i.e., true native breeds (‘Jidori’ in Japanese), and ‘Zairaisyu’ as defined in the Jidori JAS [9]. In this review, I refer to the breeds defined in the Jidori JAS as ‘Zairaisyu’.

According to the Jidori JAS [5], the percentage of Zairaisyu blood in chickens to be marketed as certified Jidori JAS must be 50% or more. It is assumed that the purpose of the Zairaisyu definition is to allow a wider range of chicken breeds to be commercially available. In addition, chickens must be reared free-range for at least 75 days at a maximum stocking density of 10 birds per square meter. Moreover, producers of Jidori JAS-certified chicken must buy chicks from hatcheries for each new production cycle, ensuring consistent production and high meat quality.

### 3.2. Flavor Profile of Jidori JAS-Certified Chickens

While the majority of Japanese consumers have a positive perception of Jidori JAS-certified chicken brands, it is important to recognize that Jidori JAS certified chickens are not certified for the flavor of their chicken meat. The evaluation of the flavor of Jidori JAS-certified chicken is a subjective matter. However, according to Takahashi (2018) [10], most Japanese consumers recognize that the meat of Jidori JAS-certified chickens has a richer flavor than that of broiler chickens. According to Rikimaru and Takahashi (2010) [11], meat texture is a key factor in consumer perception, and many Japanese consumers believe that Jidori JAS meat has a tough texture. There are several other key factors that have a significant impact on flavor perception. These include the presence of free amino acids (FAA), including glutamic acid (Glu), and purine compounds, such as inosine 5′-monophosphate (IMP). Because Glu and IMP are well-known active components of umami taste (the taste of L-glutamate), their salts have been widely used as flavor enhancers of food [12]. In Japan, studies conducted in the 1980s and 1990s compared the contents of FAA, including Glu and IMP, in the meat of Jidori JAS and broiler chickens; however, the relationship between the composition of FAA, including Glu and IMP, and the flavor of Jidori JAS chicken remained to be substantiated [11]. Concurrently, arachidonic acid (ARA), a polyunsaturated fatty acid, was identified as a possible factor influencing the flavor of JAS-certified chicken meat [11]. This was determined through a comparative analysis of biochemical components (moisture, crude protein, and crude fat), FAA, IMP, and fatty acid profile in the thigh meat of Hinai-jidori (a Jidori JAS-certified brand chicken) and broilers. Both types of chickens were raised under identical environmental conditions and fed the same diet. Based on the experiments conducted by Kiyohara et al. (2011) [13] and Takahashi et al. (2012) [14], the following was concluded: (1) the arachidonic acid (ARA) content in meat can be increased through dietary supplementation with ARA-enriched oil, and (2) meat with a high ARA content is more flavorful than meat from chickens not receiving ARA supplementation, in both Hinai-jidori [13] and broiler chickens [14]. Furthermore, Matsui and Takahashi (2017) [15] demonstrated that the ARA content in yolk is positively correlated with egg flavor. Although previous reports [13,14,15] have demonstrated a positive correlation between ARA content and sensory evaluation scores, scientists should avoid claiming that a particular brand of chicken is more flavorful only because it has a high ARA content unless they have strong experimental evidence to support such a claim.

## 4. Examples of Jidori JAS Certified Chickens

Japan is one of the well-developed countries. More than 99% of chickens on the Japanese market are broiler chickens [1]. The market share of chickens that have the Jidori JAS certificate is currently less than 1% [1]; however, Jidori JAS chicken has become well-known in each area as a local special dish. The breeding stock lines of each of the Jidori JAS chickens have been maintained and improved at the Public Institute for Poultry in each of the prefectures in Japan. There are also Jidori JAS chickens that have complete traceability systems based on the DNA discrimination method [16]. Therefore, every Jidori JAS chicken has been reared under high governance in Japan.

### 4.1. Commercial Chickens Produced by Intrabreed Strains

According to Kino (2018) [17], the Nagoya, known commercially as the Nagoya-Cochin, is a distinctive Jidori JAS chicken that has been commercialized in the Japanese market through intra-breed strain crosses. The Nagoya, a dual-purpose breed for eggs and meat, is a popular native chicken in the Aichi Prefecture, in central Honshu, Japan. In the early 1880s, local chicken around the city of Nagoya was crossed with the Chinese Buff Cochin. After that, offspring that had a buff color were selected. In 1905, during the Meiji period, the chicken was recognized as the first practical breed for poultry farming in Japan. Following the removal of the shank feathers and the fixation of the gray-colored legs, the Nagoya breed was formally established as a Japanese synthetic breed in 1919. The purebred (Nagoya × Nagoya) has been commercialized, so the commercial chickens are produced by intrastrain mating. To date, the Nagoya breed has seven strains (NGY 1 to 7), which were established at the Aichi-ken Agricultural Research Center, Nagakute, Japan. They are maintained at the Aichi Livestock and Poultry Breeding Center in Okazaki, Japan, which supplies parent stocks to the hatcheries using the seven strains. All commercial Nagoya chickens are derived from these seven strains. The market price of Nagoya meat is considerably higher than that of today’s broiler meat. In addition to meat, Nagoya eggs have been sold at a price that is two–three times higher than the standard market price in Japan. The annual production of the Nagoya-Cochin is about 840,000 birds in the fiscal year 2024 [1].

### 4.2. Commercial Chickens from F_1_ Crosses

Native Japanese chicken breeds, especially those traditionally used for fancy purposes, make it difficult to predict their growth performance for meat production. The number of eggs is also much lower than that of today’s imported breeds, as the native females retain the broodiness trait. To overcome these issues, F_1_ crosses are used to produce commercial chickens. To produce F_1_ hybrid chickens for Jidori JAS registration, dual-purpose breeds such as Rhode Island Red, White Plymouth Rock, and a synthetic breed resulting from the cross between Rhode Island Red and White Plymouth Rock are usually used as maternal lines. For paternal lines, the Hinai-dori, Shamo, and Satsuma breeds are usually used, as these breeds are historically believed to produce more flavorful chicken. Some examples of Jidori JAS chickens produced from F_1_ hybrid crosses are shown here.

#### 4.2.1. Awa-Odori Chicken

According to the Tokushima Poultry Association [18], Awa-odori is a Jidori JAS-certified brand of chicken produced in Tokushima Prefecture, which is located on Shikoku Island in southern Japan. The Tokushima Agriculture, Forestry and Fisheries Technology Support Center (Ishii-cho, Tokushima, Japan) has long improved the growth performance of large Shamo (Japanese Game) and developed a strain of Shamo called Awa-jidori. The F_1_ hybrid cross between the Awa-jidori sire and the White Plymouth Rock dam has been marketed as Awa-odori since 1990. The blood percentage of Zairaisyu in the Awa-odori chicken is 50%, since Shamo is Zairaisyu in Jidori JAS. According to [18], these chickens are fattened for 75 days or more in a comfortable and natural environment. It has been the best-selling Jidori JAS branded chicken since 1998. The annual production of Awa-odori is about 1,700,000 birds in the fiscal year 2024 [1].

#### 4.2.2. Hinai-Jidori Chicken

According to Rikimaru and Takahashi (2007) [16], Hinai-dori is a breed of chicken native to Akita Prefecture in northern Honshu, Japan. The flavor of Hinai-dori meat is well known and has been used for many years as an ingredient in the prefecture’s local dish, Kiritanpo stew. The population of Hinai-dori decreased due to competition from exotic breeds that were imported during the Meiji period (1868–1912). For a time, the breed faced the very real risk of extinction. In 1942, the Hinai-dori was designated a national treasure of Japan. According to Hatakeyama et al. (1978) [19], in order to develop Jidori JAS using Hinai-dori, the Akita Prefectural Livestock Experiment Station (Daisen, Akita, Japan) conducted single-cross tests with Hinai-dori male parents. Taste tests revealed that F_1_ meat from a cross between the Hinai-dori and Rhode Island Red breeds was best. In addition, the F_1_ individuals had a resemblance to the Hinai-dori breed. Therefore, the crossbred (Hinai-dori sire × Rhode Island Red dam) was commercialized as the Hinai-jidori chicken. Most Hinai-jidori chickens sold in the market are females. The market price of Hinai-jidori chicken meat is much higher than that of today’s broiler meat, since the fattening period is about 150 days of age. The annual production of the Hinai-jidori is about 414,000 birds in the fiscal year 2024 [1]. To date, ‘Akita capon’, the castrated Hinai-jidori chicken, is also commercially available. These male chickens are castrated by the method described by Rikimaru et al. (2011) [20].

#### 4.2.3. Amakusa Daioh Cross Chicken

According to Momoi et al. (2021) [21], the Amakusa Daioh breed, which is native to the Amakusa region of Kumamoto Prefecture, Kyushu, Japan, became extinct before World War II. The Amakusa Daioh breed was one of the largest native chicken breeds in Japan. The height and BW of the Amakusa Daioh rooster reached 90 cm and 6.7 kg, respectively. In 2001, the Amakusa Daioh breed was restored by the Animal Husbandry Research Institute, Kumamoto Prefectural Agricultural Research Center (Koshi, Kumamoto, Japan). The restoration procedure has been described in detail by Matsuzaki et al. (2001) [22]. Briefly, the Shamo, Kumamoto, and Langshan (native to China) breeds were crossed so that the blood percentages of Shamo, Kumamoto, and Langshan were 25%, 25%, and 50%, respectively, in accordance with the documented history of the establishment of the Amakusa Daioh breed. Then, closed herd breeding was conducted based on appearance, body shape, and body size. As both Shamo and Kumamoto were designated as Zairaisyu in the Jidori JAS, the blood percentage of Zairaisyu in the Amakusa Daioh breed is 50%. According to Momoi et al. (2021) [21], F_1_ hybrid chickens from the cross between Amakusa Daioh cocks and Kyushu Rhode (a synthetic breed resulting from a cross between Rhode Island Red and White Plymouth Rock) hens are available as Amakusa Daioh Cross chickens in the Japanese market. In the Kyushu Rhode, the blood percentage of Zairaisyu is 50%. Consequently, the Amakusa Daioh Cross chicken possesses 50% Zairaisyu blood. The annual production of the Amakusa Daioh Cross chicken is about 120,000 birds in the fiscal year 2024 [1].

#### 4.2.4. Kuro Satsuma-Dori Chicken

Satsuma is a breed of chicken native to Kagoshima Prefecture in southern Kyusyu, Japan. The Satsuma breed has been maintained in Kagoshima Prefecture for over 800 years, primarily for cockfighting and aesthetic purposes [23]. According to Okada et al. (1984) [24], it is thought that the Satsuma breed originated from crosses between Shamo, Shokoku, and local chickens in the Kagoshima area. It is also well known for the flavor of its meat. A F_1_ hybrid cross between the Satsuma sire and the Barred Plymouth Rock dam has been marketed as Kuro Satsuma-dori chicken since 2006. The annual production of the Kuro Satsuma-dori is about 128,200 birds in the fiscal year 2024 [1].

### 4.3. Commercial Chickens from Three-Way Crosses

#### 4.3.1. Miyazaki Jitokko Chicken

According to Horinouchi et al. (2019) [25], the Jitokko breed, which was declared a natural treasure in 1943, has been maintained at the foot of Mt. Kirishima, located between Miyazaki and Kagoshima Prefectures in southern Kyushu Island, Japan. The characteristics of the breed are Jidori-type plumage, short legs, large crests, and a beard. The short-leg trait in the Jitokko breed is controlled by a dominant lethal gene, Creeper (*Cp*), which is manifested as short legs in heterozygous (*Cp*/*+*) chickens and embryonic lethality in homozygous (*Cp*/*Cp*) embryos [26]. Jitokko hobbyists, even in the absence of knowledge on heredity, have long selected for and maintained birds with short legs. In the Kawaminami Branch of the Miyazaki Prefectural Livestock Research Institute (Kawaminami-cho, Miyazaki, Japan), studies on producing a new Jidori brand of chicken utilizing the Jitokko breed as a founder began in 1985. Miyazaki Jitokko is a three-way crossbred chicken produced by crossing F_1_ Jitokko sire cocks (*Cp*-free (+/+) with White Plymouth Rock dams) and Kyushu Rhode hens. Miyazaki Jitokko chickens have been marketed since 1990. The annual production of the Miyazaki Jitokko is about 261,000 birds in the fiscal year 2024 [1].

#### 4.3.2. Meat-Type Okumino-Kojidori Chicken

According to Ishikawa et al. (2020) [27], Gifu-jidori is a Jidori variety native to the Gifu Prefecture, an inland prefecture located in the center of Honshu, Japan. The meat-type Okumino-kojidori chicken is a commercial brand of chicken produced in the Gifu Prefecture. The meat-type Okumino-kojidori chicken is a three-way hybrid chicken produced by crossing Gifu-jidori improved breed cocks, F_1_ hybrid hens of a White Plymouth Rock cock, and a Rhode Island Red hen. The Gifu-jidori improved breed is a synthetic developed at the Seki Experiment Station, Department of Swine and Poultry Science, Gifu Prefectural Livestock Research Institute (Seki, Gifu, Japan). Four breeds, Gifu-jidori, Red Cornish, New Hampshire, and Red Rock, were crossed to produce the Gifu-jidori improved breed. The Gifu-jidori improved breed has been maintained to be a hereditary percentage of the Gifu-jidori breed by more than 50%. The annual production of the meat-type Okumino-kojidori is about 78,530 birds in the fiscal year 2024 [1].

## 5. Current Research Activities Using Jidori and Jidori JAS-Certified Chickens

### 5.1. Marker-Assisted Selection for Improvement of Growth Performance

#### 5.1.1. QTL Identification of a Candidate Gene for Improving Growth Traits in Hinai-Dori Breed

According to Rikimaru et al. (2011) [28], since 1973, when fertilized eggs from the Preservation Society (PS) of the Hinai-dori breed were introduced to the Akita Prefectural Livestock Experiment Station (LES), LES has been conducting selection experiments to improve growth performance. According to Rikimaru et al. (2013) [29], the average body weight at 14 weeks of age is 2023.9 ± 284.5 g for LES, whereas the average body weight at 14 weeks of age is 1199.4 ± 211.4 g for PS. As the difference in growth performance, such as body weight, between PS and LES lines is the result of long selection over 30 years, it is suggested that these individuals possess genes that influence growth traits. To efficiently detect quantitative trait loci (QTL) for growth traits within the Hinai-dori breed, an F_2_ resource population was developed by crossing PS sires with LES dams, and thereafter, QTL mapping was conducted [28]. As a result, three QTL regions, two distinct QTLs on chromosome 1 and one QTL on chromosome 4, affecting growth traits were successfully detected in the resource population [28]. Regarding to the QTL on chromosome 4, Rikimaru et al. (2013) [29] assumed that cholecystokinin type A receptor (*CCKAR*) is a candidate gene for growth traits because it was located just under the QTL peak on chromosome 4 and has also been identified as a candidate gene for human obesity [30]. Accordingly, Rikimaru et al. (2013) [29] analyzed polymorphism in the *CCKAR* gene and tested its association with growth traits in the F_2_ population. As a result, five *CCKAR* haplotypes were identified, and significant associations were observed between *CCKAR* haplotypes and growth traits. Furthermore, Rikimaru et al. (2013) [29] found a single-nucleotide polymorphism (SNP) (A/C) in the predicted Yin Yang 1 (YY1) binding site in the 5′-untranslated region (5′-UTR) of the *CCKAR* gene (SNP, AB604331: g.420 C>A), and the haplotype data implied that this SNP might be associated with growth traits. At that time, a significant association between the SNP and growth traits was identified in the Hinai-dori breed (paternal founder of the Hinai-Jidori breed); however, the practical application of the SNP for marker-assisted selection to improve growth traits remains to be seen in commercial Jidori JAS chickens, including Hinai-jidori chickens.

#### 5.1.2. Marker-Assisted Selection for Improvement of Growth Performance in Jidori JAS Chickens

To verify whether the g.420 C>A SNP in *CCKAR* found in Hinai-dori can be used as a marker to improve the growth characteristics of Jidori JAS-certified chickens, further trials were carried out at public institutes in Akita, Gifu, Kumamoto, and Miyazaki prefectures. All parent stock lines of Hinai-jidori (Akita Prefecture), Okumino-kojidori (Gifu Prefecture), Amakusa Daioh Cross (Kumamoto Prefecture), and Miyazaki-jitokko (Miyazaki Prefecture) chickens were selected by using the A-allele of the SNP as a molecular marker, and resultantly A-allele fixed lines were developed. From the A-allele fixed lines, four Jidori brand chickens were practically produced as their improved types. Their growth performance was then compared with that of conventional chickens. As a result, a significant improvement in slaughter live weight was observed in the Jidori brand chickens ([21], Amakusa Daioh Cross chickens; [31], Miyazaki Jitokko chickens; [27], meat-type Okumino-kojidori chickens; [32], Hinai-jidori chickens).

After we published these sequential manuscripts, an exception was reported by Kawae and Mitani (2020) [33], who found that A-allele fixation did not improve growth traits in the meat-type Sanuki Cochin chicken, a Jidori JAS chicken produced in Kagawa Prefecture. The meat-type Sanuki Cochin is an F_1_ hybrid cross between the Sanuki Cochin (a local variety of Chinese Buff Cochin) sire and the White Plymouth Rock dam. Meanwhile, the Hinai-jidori, Okumino-kojidori, Amakusa Daioh Cross, and Miyazaki-jitokko chickens use Rhode Island Red or Rhode Island Red derivatives as maternal lines. The use of fast-growing White Plymouth Rock as the maternal line may obscure the improvement effect of the *CCKAR* SNP.

### 5.2. Utilization of Native Chickens in Research Contexts

As previously mentioned, Japanese native chickens are diverse in appearance and characteristics and are unique compared to chickens from other countries. In other words, Japanese native chickens are a treasure trove of rare chicken genetic resources by global standards and excellent research material for elucidating the genes responsible for their characteristic traits.

The Chabo (Japanese Bantam) breed is designated as one of the national treasures of Japan. The ancestors of the Chabo are thought to have been introduced to Japan from Vietnam around the beginning of the 17th century [2]. The Chabo has a small body, a pretty shape with upright tail feathers, and its character is friendly and gentle. The Chabo also possesses the Creeper (*Cp*) trait, as was outlined in the introduction to the Jitokko breed. Kinoshita et al. (2020) [34] reported that a 25kb deletion on chromosome 7, which contains *Indian hedgehog* (*IHH*) and *non-homologous end-joining factor 1* (*NHEJ1*) genes, is responsible for the Creeper-trait. *IHH* is essential for chondrocyte maturation and is downregulated in the *Cp*/*+* embryos and is completely lost in the *Cp*/*Cp* embryos. This indicates that chondrodystrophy is caused by the loss of *IHH* and that chondrocyte maturation is delayed in *Cp*/*+* heterozygotes [34].

The Uzura chabo (Uzurao for short), a variety of the Chabo breed, exhibits a distinctive characteristic known as ‘Rumpless (*Rp*)’. This results in the absence of an observable tail. Since this trait is known to be caused by an autosomal dominant, non-lethal gene, the Uzura chabo is thought to have a fixed genotype of *Rp*/*Rp*. Regards to the Rumpless-trait, Guo et al. (2023) [35] reported that (1) through genome-wide association and linkage analyses using the Piao chicken, native to China, the candidate region was fine-mapped to 798.5 kb in the central region of chromosome 2 (chr 2: 86.9 to 87.7 Mb), (2) Explorations of the expression data identified a novel causative gene, *Rum*, that produced a long, intron less transcript across the deletion, and (3) the expression of *Rum* is embryo-specific, and it regulates the expression of *MSGN1*, a key factor in regulating T-box transcription factors required for mesoderm formation and differentiation. These results provide genetic and molecular experimental evidence for a mechanism that regulates tail development in chickens [35], but further studies are needed to determine whether the same mechanisms apply to other rumpless chickens, including the Uzura chabo.

Males of the Tosa Onaga-dori (Japanese Long Tail) show no molting in some tail feathers and saddle hackles throughout their lives [2]. According to Matsuo (2024) [36], long-tailed chickens were introduced to Japan via the Korean peninsula by the 8th century. Recently, a suggestive manuscript on the genetic causes of the long-tailed trait has been reported using the Korean long-tailed chicken, ‘Ginkkoridak’ in Korean [37]. According to [37], (1) a number of selected genomic loci related to long tail feathers were found in KLC, (2) the genomic regions showing significantly high copy numbers in KLC were found, and (3) these include genomic regions harboring a cluster of feather keratin 1-like genes. If the Tosa Onaga-dori and the Ginkkoridak are from the same genetic lineage, the genetic causes why the Tosa Onagadori has a long tail may be discovered.

## 6. Governance to Maintain Native Chicken Breeds in Japan

One can assume that native breeds of chicken in Japan are distinguished according to the two purposes for which they are maintained, one for hobbyists for fun and the other for commercial production. As relatives of poultry breeders and producers know, today’s chickens are at constant risk of epidemics, especially avian influenza in the winter season. In Japan, hobbyists mainly keep native breeds of chickens in small populations in remote areas. Due to the fact that these populations are isolated from the outside world, the risk of influenza outbreaks is minimal. However, there is a risk of attack by wild animals such as foxes, raccoons, and wild dogs.

Japan has chosen the route of allowing commercial use of its hybrids under the Jidori JAS [5], rather than recommending and enforcing commercial use of the native chickens themselves. Consequently, native chickens are not only kept in public research institutes in situ with hygienic conditions, but also continuously used to produce hybrid chickens. Japan is considered one of the successful countries in terms of effectively using and preserving native chickens.

### 6.1. National Genebank Project Regarding Chickens Native to Japan

In the event of a threat to the extinction of Japanese chickens, 14 breeds, as shown in Table 3, are now preserved as frozen semen at NARO, Japan, as part of a national Genbank project administered by MAFF [38]. In addition, NARO has collected primordial germ cells (PGCs) for cryopreservation and has successfully regenerated Gifu-jidori [39] and Hinai-dori [40] from cryopreserved PGCs.

### 6.2. Rules for Sanitation and Veterinary Medicine for Highly Pathogenic Avian Influenza in Japan

According to the MAFF of Japan [41], if there is an outbreak of highly pathogenic avian influenza in Japan, all poultry on the affected farm will be killed quickly and their carcasses buried or incinerated. Anything on the farm will also be incinerated, buried, or disinfected. Chicken coops will also be disinfected. As a general rule, the movement of poultry and other animals is prohibited within a 3 km radius of the affected farm (movement restriction area). In addition, within a 10 km radius, movements of live poultry within the area and movements from outside the area into the area are allowed, but the transport of poultry and other animals outside the area is prohibited (transport restriction area). These movement and transport restriction areas shall be lifted following a negative result in a confirmatory avian influenza test carried out at the end of the quarantine period. In short, the Japanese government has adopted a policy that the best way to contain avian influenza is to take all possible measures to prevent the spread of the virus, such as culling all infected chickens and disinfecting poultry farms. Fortunately, Japan has not historically experienced a simultaneous nationwide spread of avian influenza, and outbreaks have remained sporadic.

According to Haque et al. (2024) [42], due to a highly pathogenic avian influenza outbreak in 2014, all Korean native chickens restored breeds at the Poultry Department of the Institute of Animal Science were culled. The extant restored breeds of Korean native chickens are descended from a partially dispersed species at the Namwon Livestock Genetic Resources Center [42]. In certain countries, the practice of culling infected poultry is not a viable option. In China, Vietnam, Indonesia, and Egypt, vaccination of poultry is employed as an alternative to culling. However, despite the fact that vaccinated chickens exhibit milder symptoms, they nevertheless retain the capacity for viral transmission, a factor which can result in the emergence of “hidden infections” [43]. It is important to prevent the spread of infectious diseases in a way that is appropriate for each country.

### 6.3. Is Jidori JAS System Applicable in Other Countries?

The answer to this question depends on the situation of poultry production in each country. For example, in Thailand, the population of native chickens in 2024 was 114,939,469, while the number of native chicken farmers was 2,764,104 [44]. The mean number of chickens per farmer is calculated to be 41.6. In Thailand, most local chickens are raised in backyards and fed local grains and food scraps [44]. They are bred naturally without specific management, which results in crossbreeding and the potential loss of purebred characteristics [44]. For example, in Kenya, native chickens are about 22 million and kept by 90% of the rural communities in small flocks of up to 30 birds under a free range system [45]. The average number of chickens kept per farmer is 41.6 in Thailand and 30 in Kenya, which is comparable to the number typically maintained by native chickens per hobbyist in Japan. According to the data set provided by [1], the average number of chickens produced per producer in the fiscal year 2024 is estimated as follows: 32,692 birds for Awa-Odori, 21,556 for Nagoya-Cochin, 5750 for Hinai-Jidori, 17,143 for Amakusa Daioh Cross, 8547 for Kuro Satsuma-Dori, 8700 for Miyazaki Jitokko, and 15,707 for Okumino-Kojidori. Jidori JAS-certified chicken producers in Japan raise considerably higher numbers of birds than their counterparts in Thailand and Kenya. In addition, fattening chicks of Jidori-certified JAS chickens are provided by hatcheries. Natural breeding of chickens on each producer’s farm is not permitted. Therefore, it can be assumed that implementing a system similar to the Japanese Jidori JAS system in developing countries is not currently a viable option. Meanwhile, due to their experience with intensive poultry operations, nations belonging to “emerging markets and developing economies” like China may adopt a system comparable to Japan’s Jidori JAS system. Ultimately, it is up to individual governments to establish a legal framework or governance system that aligns with the practical needs and circumstances of their provinces and nations.

Jidori JAS-certified chickens sell for two to five times more than regular broilers in Japan, because such birds require a relatively long grow-out time, the production cost increases considerably [11]. In Japan, Jidori JAS-certified chickens are not consumed daily, but rather served as special dishes to entertain guests, celebrate happy occasions, or enjoyed as regional delicacies while traveling. It is imperative to ensure a sufficient number of consumers willing to purchase such high-priced chicken to maximize sales potential. Consequently, it is logical to infer that high-quality chickens could be purchased by China’s high-income earners, creating a lucrative market opportunity.

## 7. Conclusions

In conclusion, the current status of the conservation and commercial use of the chicken breeds native to Japan has been summarized. Japan possesses a rich array of genetic resources for chickens. Furthermore, Japan, being an island nation, does not have any direct land border with any other country. This geographical reality makes it easy and advantageous from a geopolitical perspective to prevent the influx of infectious diseases from overseas. Given that the maintenance of many native chicken breeds has been relegated to hobbyists, the need for public support for the conservation of these breeds is a likely eventuality in the future. However, it is highly unlikely that the breeds frequently used to produce Jidori JAS chickens, such as Hinai-dori, Shamo, and Satsuma, will become extinct. It is hoped that other countries will find the information on the conservation of genetic resources in Japan useful.

## Figures and Tables

**Table 1:** List of native chicken breeds of Japan that have been designated as national treasures of Japan.

Ukokkei, Koeyoshi, Shamo, Tomaru, Minohiki, Uzura-chabo, Satsuma, Shokoku, Tosa Onaga-dori, Kawachi-yakko, Jitokko, Chabo, Hinai-dori, Kuro-kashiwa, Jidori, Totenko, Minohiki

**Table 2:** List of Zairaisyu chicken breeds in Jidori JAS.

Category	Purpose	Breed
1. Breeds established prior to the close of the Edo period	Fancy	Gifu-jidori, Ise-jidori, Tosa-jidori, Tsushima-jidori, Iwate-jidori, Aizu-jidori, Hinai-dori, Gan-dori, Shibbatori, Sado-hige-jidori, Jitokko, Satsuma, Kawachi-yakko, Kuro-kashiwa, Kureko-dori, Chabo, Uzura-chabo, Minohiki-chabo, Minohiki, Shokoku, Tosa Onaga-dori, Ukokkei, Ingie, Utaicharn, Okinawa-hige-jidori *
	Fancy + Long-crowing	Tomaru, Koeyoshi, Totenko
	Fancy + Cock-fighting	Shamo
2. Synthetic breeds crossing Japanese local chickens and breeds imported from foreign countries	Fancy	Miyaji-dori
	Utility	Nagoya, Mikawa, Eikoku, Kumamoto, Tosa-kukin
3. Breeds imported from foreign countries	Utility	Barred Plymouth Rock, Rhode Island Red, Chinese Buff Cochin

* According to Minezawa (2005) [8], there is currently no evidence to suggest whether Okinawa-hige-jidori is present. According to [8], the president of the Utaicharn Preservation Society hinted that there was a possibility that a Utaicharn cross would be registered as an Okinawa-hige-jidori.

**Table 3:** List of breeds conserved in NARO as frozen semen.

			Category	
	Japanese Name	English Name *^1^	Jidori *^2^	Zairaishu *^3^
1	Kinpa	Japanese Henny Feathered Game	○	
2	Gifu-jidori	Japanese Old Type-Gifu	○	○
3	Tosa-jidori	Japanese Old Type-Tosa	○	○
4	Hinai-dori	Japanese Dainty	○	○
5	Koeyoshi	Japanese Good Crower	○	○
6	Tomaru	Japanese Black Crower	○	○
7	Shokoku	Japanese Elegancy	○	○
8	Satsuma	Kagoshima Game	○	○
9	Shamo	Japanese Game	○	○
10	Ukokkei	Japanese Silkie	○	○
11	Nagoya			○
12	Kumamoto			○
13	Tosa-kukin			○
14	Barred Plymouth Rock			○

*^1^ English name taken from [3]. *^2^ Japanese native breeds of chickens, which were established before the end of the Edo period (1603–1868). *^3^ Japanese chicken breeds that were established in Japan and/or imported prior to the close of the Meiji period (1868–1912), designated by Jidori JAS. The circles indicate whether each breed belongs to one or both of the two categories, Jidori and Zairaisyu.

## Data Availability

The data supporting the review are available from the corresponding author, H.T., upon reasonable request.

## Abbreviations

The following abbreviations are used in this manuscript:

NARO	National Agriculture and Food Research Organization
JAS	Japan Agriculture Standards
MAFF	Ministry of Agriculture, Forestry and Fisheries
*Cp*	Creeper gene
PS	Preservation Society
LES	Livestock Experiment Station
QTL	Quantitative trait loci
CCKAR	Cholecystokinin type A receptor
SNP	Single-nucleotide polymorphism
YY1	Yin Yang 1
5′-UTR	5′-Untranslated region
*IHH*	Indian hedgehog gene
*NHEJ1*	Non-homologous end-joining factor 1 gene
*Rp*	Rumpless gene
*MSGN1*	Mesogenin 1 gene
KLC	Korean long-tailed chicken
